# The About 100 Million Years Old Enigmatic “Beak Larva” Is a Weird Click Beetle Relative

**DOI:** 10.3390/insects17030316

**Published:** 2026-03-14

**Authors:** Simon J. Linhart, Carolin Haug, Jörg U. Hammel, Sabine Saß, Joachim T. Haug

**Affiliations:** 1 Biocenter, Ludwig-Maximilians-Universität München (LMU Munich), Grosshaderner Str. 2, 82152 Planegg, Germany; simon.linhart@palaeo-evo-devo.info (S.J.L.); carolin.haug@palaeo-evo-devo.info (C.H.);; 2 GeoBio-Center, Ludwig-Maximilians-Universität München (LMU Munich), Richard-Wagner-Str. 10, 80333 München, Germany; 3 Institute of Materials Physics, Helmholtz-Zentrum Hereon, Max-Planck-Str. 1, 21502 Geesthacht, Germany

**Keywords:** Elateroidae, Jurasaidae, Cerophytidae, Cretaceous, Kachin amber

## Abstract

“Beak larva” is a collective term for four fossil specimens from Kachin amber, which are holometabolan larvae with a forward-projecting beak. This arrangement is very unusual, and so far it has been unclear whether these specimens are larvae of beetles or of lacewings. We present here new details of the mouthparts of one of these larvae based on synchrotron radiation-based X-ray µ-CT (SRµCT) imaging. We can identify that the main part of the beak is formed by the labrum and the labium; the mandibles insert laterally into this beak. The beak has two distinct channels to which the mandibles seem to be connected. The maxillae are tightly connected to the labium and head capsule and have an endite each (unclear if lacinia, galea, or mala), also inserting into the beak. Overall, these details reveal a mouthpart arrangement incompatible with an interpretation as a lacewing. The arrangement is most similar to that in some beetle larvae, namely those of Elateroidea. It most closely resembles that of the larvae of the species-poor sister groups Cerophytidae and Jurasaidae. It seems likely that the beak larvae are closely related to Jurasaidae, possessing an intermediate morphology. The combined data allow us to reconstruct aspects of the entire group, including the beak larvae, Cerophytidae, and Jurasaidae, indicating that they undergo hypermetamorphosis. The latter is most expressed in Jurasaidae. Combined with the paedomorphic female, this group seems to have four rather distinct morphologies and ecologies: (1) early mobile larvae, (2) physogastric, strongly wormlike larvae, (3) “normal”-appearing adult males, and (4) females with another unique morphology.

## Simple Summary

“Beak larva” is a term used for four fossil larvae with specific mouthparts resembling a beak. These mouthparts are rather untypical, and it is unclear whether the larvae are lacewings or beetles. We present new details of one beak larva, scanned with synchrotron radiation. We can identify the composition of the beak from the position of the different structures of the mouthparts. The arrangement of the mouthparts opposes the interpretation of the larva as a lacewing and is most similar to mouthpart arrangements in the beetle group Elateroidea. Within the latter, the closest similarity is with the species-poor sister groups Cerophytidae and Jurasaidae. It seems most likely that the beak larvae are part of the group Cerophytidae + Jurasaidae, with an intermediate morphology between Cerophytidae and Jurasaidae. With the accessible data, we are able to reconstruct aspects of the whole group, also indicating that they undergo a special developmental pattern described as hypermetamorphosis. In Jurasaidae, this pattern is most expressed, with four distinct morphologies and ecologies. These are an early mobile larva, a wormlike larva with an inflated trunk, “normal”-appearing adult males, and adult females with another unique morphology.

## 1. Introduction

Extinct animals in the fossil record sometimes puzzle us due to their bizarre morphologies, which makes it challenging for us to evaluate who could be the next living relative. Famous examples of extreme cases include *Anomalocaris* [1] or *Tullimonstrum* [2]. Yet, also on a smaller scale, both in body size and the range of possible phylogenetic relationships, there are numerous such examples. Maybe only a minor case, but a still interesting one, is that of the so-called “beak larva”.

Haug et al. [3] reported the first beak larva, a small holometabolan larva from about 100-million-year-old Kachin amber, in Myanmar. It had an unusual combination of features that led them to conclude that it might be a larva of a beetle (Coleoptera), but that it could also possibly be the larva of a lacewing (Neuroptera). The mouthparts of the larva formed a functionally forward-directed beak, leading to the nickname “beak larva”. While functional beaks are well known in non-holometabolan immatures, for example, in hemipterans [4] and adult holometabolans, such as mosquitoes [4], this kind of mouthpart morphology is highly unusual for a holometabolan larva. As a possible comparison, beetle larvae of Ceryloninae also have a beak, but it is backward-oriented [5]. However, in some lacewings, the two compound jaws or stylets (formed by each mandible and its corresponding maxilla) can also be tightly held together, appearing like a single forward-directed beak [6]. The beak larva of Haug et al. [3] was not well enough preserved to resolve the details of the mouthparts and allow a more detailed comparison of the structures forming the beak, hence leaving the question of a possible closer relative (beetle or lacewing) unanswered. Due to its unique morphology, the larva was formally described as *Partisaniferus atrickmuelleri*.

Additional specimens from the same fossil fauna became available, possessing the same types of mouthparts but differing in the trunk morphology [6,7,8]. Based on the differences, a second species was formally described: *Partisaniferus edjarzembowskii*. Of this species, three ontogenetic stages could be differentiated, showing that the later larvae were physogastric and that the larvae were likely wood-associated. Some aspects of the reconstructed ontogeny indicated that *Partisaniferus* was more likely a beetle than a lacewing; however, none of the new specimens provided further distinct clues from the mouthparts.

Unnoticed by the authors of the beak larva, another strange larva of an extant beetle was reported in the same year as the first beak larva. Rosa et al. [9] reported Jurasaidae, a new ingroup of Elateroidea, the click-beetle-like forms. The supplement of Rosa et al. [9] depicted a single physogastric larva, which also had mouthparts forming a forward-directed beak, together with details of more beak-bearing larvae.

We here provide new details of the mouthparts of a larva of *P. edjarzembowskii*, a beak larva, and compare it to the larvae of Jurasaidae and their closer relatives. In this way, we provide a better-founded interpretation of the fossil larva, but also aspects of the evolution of larvae and ontogeny within a peculiar evolutionary lineage of Elateroidea.

## 2. Materials and Methods

### 2.1. Materials

A single specimen was directly studied. All other resources are based on data from the literature. The single specimen is PED 0596 and was already reported in Haug et al. [6]. It is embedded in Cretaceous Kachin amber from the ~99-million-year-old deposits of Hukawng Valley, Kachin State, Myanmar [10,11,12]. It is part of the Palaeo-Evo-Devo Research Group Collection of Arthropods, Ludwig-Maximilians-Universität München, Germany. The specimen was legally purchased on eBay.com, from the trader burmite-miner.

### 2.2. Documentation Method

Specimen PED 0596 was first documented on a Keyence VHX-6000 digital microscope (Keyence, Osaka, Japan). Illumination was transmitted light; compound imaging (stacking, fusion, stitching, HDR) was applied.

Specimen PED 0596 was subsequently scanned at the Imaging Beamline P05 [13,14], operated by the Helmholtz-Zentrum Hereon at the PETRA III storage ring (Deutsches Elektronen Synchrotron—DESY, Hamburg, Germany). Imaging was performed at a photon energy of 20 keV and a sample-to-detector distance of 60 mm. Projections were recorded using a custom-developed 20 MP CMOS camera system [15] with an effective pixel size of 0.64 µm. For each tomographic scan, 3001 projections at equal intervals between 0 and π were recorded. Tomographic reconstruction was done by applying a transport of intensity phase retrieval approach and using the filtered back projection algorithm (FBP) implemented in a custom reconstruction pipeline [16] using Matlab (version: 24.2.0.2863752 (R2024b) Update 5, Math-Works Inc, United States, Massachusetts, Natick, https://github.com/moosmann/matlab (accessed on 9 March 2026) Git commit ID: Mem: 4315807047680 1555075956736 803313885184 801669120 1977962549248 2760731090944) and the Astra Toolbox (open access) [17,18,19]. For the processing, raw projections were binned two times for further processing, resulting in an effective pixel size of the reconstructed volume of 1.33 µm. Other specimens of the beak larva did not provide informative scans.

### 2.3. Image Processing

The TIFF stack resulting from the synchrotron scan was further processed in different programs. FIJI (open access) was used to crop the stack and orient it in standard directions. Regions of interest were cropped out and further dealt with separately due to the rather large size of the original scan. The resolution of the scan was still not good enough to properly segment all parts in the beak from the individual frames of the stack, as they formed a rather uniform white area. Some cavities could be recognised and were segmented using Drishti Paint (open access). The TIFF stack was first oriented perpendicular to all major axes using FIJI. The stack was then imported using Drishti Import v2.6.4 (open access), and structures of interest were manually segmented in Drishti Paint. Drishti files were imported into Meshlab (open access). Based on these, obj files were exported and rendered in Blender 2.48 (open access).

In addition, stereo anaglyphs of volume renders were produced in Osirix (open access). These were red-blue anaglyphs and were transformed into red-cyan anaglyphs using Adobe Photoshop CS2 (Version 9.0, Adobe Systems Inc., San José, CA, USA). Based on these, the structures were traced in Adobe Photoshop CS2.

### 2.4. Measurements

Length and width were measured in dorsal view based on extant and fossil larval and adult specimens from the literature (Supplementary Table S1). Images were magnified on the screen and then measured with a caliper. Based on these values, ratios were calculated.

## 3. Results

### Interpretation of Observed Structures

**Figure 1:**
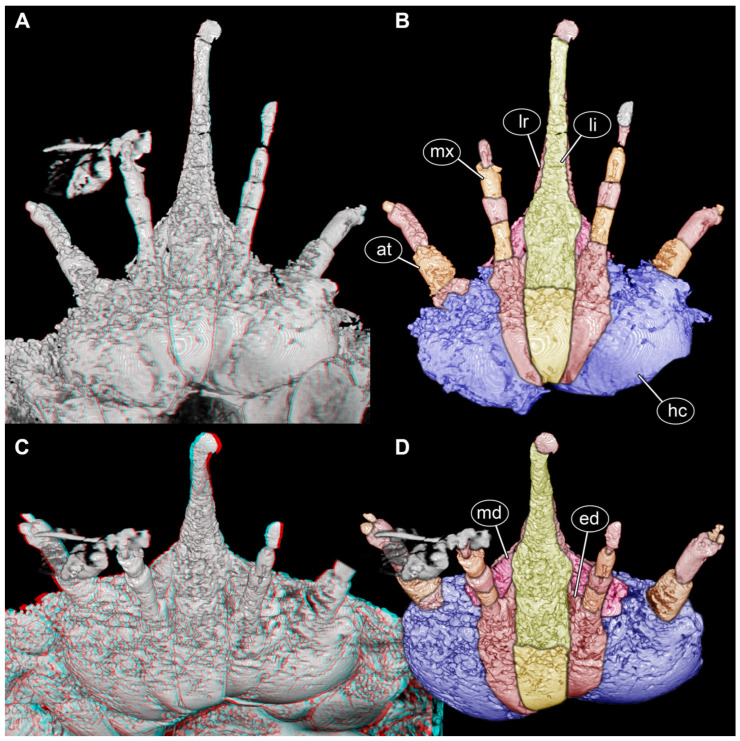
Volume renderings of the scan of the head region of a beak larva, PED 0596; note: no scale is provided due to the strong perspective of the 3D representation. (**A**,**B**) Ventral view. (**A**) Red-cyan stereo anaglyph, please use red-cyan glasses to view. (**B**) Colour-marked version of (**A**). (**C**,**D**) Antero-ventral view. (**C**) Red-cyan stereo anaglyph, please use red-cyan glasses to view. (**D**) Colour-marked version of (**C**). Abbreviations: at = antenna; ed = endite; hc = head capsule; li = labium; lr = labrum; md = mandible; mx = maxilla.

As is known from earlier observations, the anterior region of the head is drawn out into a beak-like structure. A pair of antennae and a pair of palps are also apparent (Figure 1A,B).

In the ventral view (Figure 1A,B) and antero-ventral view (Figure 1C,D), a clear subdivision of the ventral area of the head region is apparent. A distinct middle region continuing into the beak region is interpreted as the labium, although it remains unclear if parts of a possible hypopharynx contribute to this structure. Proximally, a distinct sclerite is recognisable, which may represent a prementum or a gula.

Proximally left and right of the labium, another sclerite is apparent. It is only weakly separated from the further lateral region, but interpreted as the proximal part of the maxilla (continuous stipes and cardo?). Distally, the two apparent palps insert, identifying them now clearly as palps of the maxilla.

**Figure 2:**
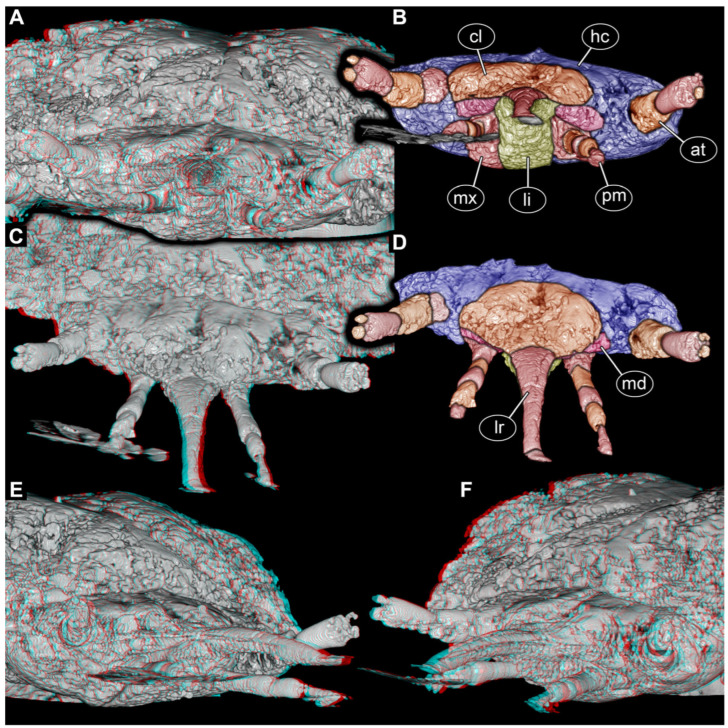
Volume renderings of the scan of the head region of a beak larva, PED 0596, continued; note: no scale is provided due to the strong perspective of the 3D representation. (**A**,**B**) Anterior view. (**A**) Red-cyan stereo anaglyph, please use red-cyan glasses to view. (**B**) Colour-marked version of (**A**). (**C**,**D**) Antero-dorsal view. (**C**) Red-cyan stereo anaglyph, please use red-cyan glasses to view. (**D**) Colour-marked version of (**C**). (**E**,**F**) Antero-lateral views, red-cyan stereo anaglyph, please use red-cyan glasses to view. (**E**) Right side. (**F**) Left side. Abbreviations: at = antenna; cl = clypeus; hc = head capsule; li = labium; lr = labrum; md = mandible; mx = maxilla; pm = palp of maxilla.

Medially to the palp, a short endite is apparent in frontal (Figure 2A,B) and antero-lateral views (Figure 2E,F). It remains unclear if this structure represents galea, lacinia, or a compound of both (mala). Further laterally, the sclerites are the ventral parts of the head capsule.

The functional ventral part of the beak (posterior in evolutionary axis) formed by the labium is separated by a distinct furrow from the functional dorsal part, barely visible in the ventral (Figure 1A,B) and antero-ventral views (Figure 1C,D) but well apparent in the anterior view (Figure 2A,B) and antero-lateral view (Figure 2E,F). The dorsal part is interpreted as the labrum that is weakly offset from the more proximal clypeus (Figure 2C,D); it is unclear whether other sclerites also contribute to this region. The clypeus is even less distinctly separated from the head capsule (Figure 2C,D).

**Figure 3:**
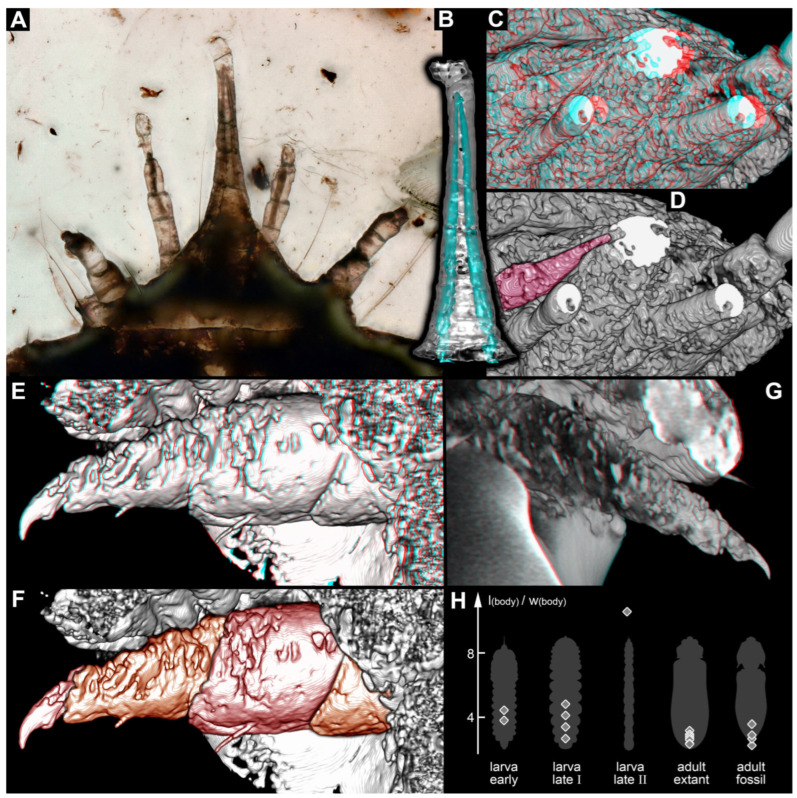
More details of the beak larva and quantitative comparison. (**A**) Compound light micrograph (transmitted light), dorsal view; note the visible channels in the beak and palps of the maxilla. (**B**–**G**) Rendering of scan; note: no scale is provided due to the strong perspective of the 3D representation. (**B**) Surface model of the distal part of the beak with the two channels in green. (**C**–**G**) Volume renderings. (**C**,**D**) Head in antero-lateral view, distal parts cut off, note the channels in the beak and palps of the maxillae. (**C**) Red-cyan stereo anaglyph, please use red-cyan glasses to view. (**D**) Simple version of (**C**) with mandible colour-marked, revealing how its distal recognisable part reaches into one of the channels of the beak. (**E**,**F**) Right foreleg. (**E**) Red-cyan stereo anaglyph, please use red-cyan glasses to view. (**F**) Simple versions of (**E**) with four visible leg elements marked; note how deeply immersed the proximal part is in the central body. (**G**) Left foreleg, red-cyan stereo anaglyph, please use red-cyan glasses to view. (**H**) Scatter plot of the body ratios for different ontogenetic stages of Cerophytidae + (*Partisaniferus* + Jurasaidae); early larvae are exclusively fossil; late larvae I are those of Cerophytidae and *Partisaniferus*; the late larva II is one of Jurasaidae; extant adults include those of Cerophytidae and Jurasaidae; fossil adults have been attributed to Cerophytidae; outlines are from various sources, from left to right: *Partisaniferus atrickmuelleri* [3] (figure 4A p. 13); *Cerophytum elateroides* [20] (figure 10A p. 384); *Jurasai itajubense* [9] (supplementary figure 12d supplementary p. 34); *Cerophytum lii*, female [21] (figure 1B p. 4); *Necromeropsis minutus* [22] (figure 12A p. 67).

Distally, the labrum appears to have a kind of lobe. While this resembles some of the artefacts around the palps, it is also well apparent in light microscopy (Figure 3A) and appears to be a real structure.

Proximally, the labrum and labium appear to gape to a certain degree, allowing for another structure to be squeezed in between them from left and right (Figure 2E,F). These structures are interpreted as the mandibles (Figure 1, Figure 2 and Figure 3C,D).

Inside the beak, two prominent channels are apparent (Figure 3B); after recognising them on the scan, they are also very prominent in the light micrograph (Figure 3A). Comparable channels are also apparent in the palps of the maxilla (Figure 3C,D).

The legs (Figure 3E–G) are quite stout. They seem deeply immersed in the bulging structures of the central body, concealing the proximal region.

## 4. Discussion

### 4.1. The Beak Larva Is Not a Lacewing

While two species of *Partisaniferus* are known, and the new data are only available for one of them, we still think that, due to the strong similarity in the mouthpart morphology, the same detailed structures account for the other species. The newly observed details of the mouthparts of the beak larva make it very unlikely that it represents a lacewing. In lacewing larvae, the labium is strongly integrated into the ventral side of the head capsule, and each maxilla is tightly interconnected with its corresponding mandible and is strongly shifted anteriorly without any functionally ventral parts and without palps [23,24]. This is very different from what is apparent in the fossil.

The mouthpart arrangement in the fossil is compatible with an interpretation as a beetle. The strong interconnection of labium and maxillae forming a maxillo-labial complex resembles that of different beetles, among them representatives of Cleroidea [25], Dryopoidea [26], or Scarabaeoidea [27]; the similarity, however, is highest to larvae of Elateroidea [28]. The latter includes click beetles in the strict sense (Elateridae) and many click beetles in the wider sense (Cerophytidae = rare click beetles; Eucnemidae = false click beetles), but also many beetles with bioluminescence and/or paedomorphic females [29].

The legs, which are well accessible at least in the single specimen of *P. atrickmuelleri*, are also compatible with such an interpretation due to their number of elements. Similar to other larvae of Polyphaga, *P. atrickmuelleri* has five prominent leg elements [3].

### 4.2. Similarities of the Fossil with Certain Larvae of Elateroidea

Within Elateroidea, the few known larvae of Jurasaidae are known to have a distinct forward-oriented beak. The main part of this beak is formed by the labium and associated structures in the larva of *Jurasai itajubense* [9] (supplementary figure 10a,d). Also, the labrum is drawn out, projecting forward, in this aspect resembling the fossil; however, the labrum is here not tightly interconnected with the beak. Furthermore, the mandible is integrated in the mouthpart complex in *J. itajubense* [9] (supplementary figure 10a,d) and in the fossil (Figure 2E,F). Two distinct channels are also present in both; in the extant larva, the mandibles protrude into these channels [9] (supplementary figure 12e). This detail could not be fully resolved in the fossil, but the channels are well apparent (Figure 3A–D), and the mandible is at least inserting into the proximal part (Figure 3C,D). The further distal part of the mandible, potentially extending into the channel, may have been simply too thin to be resolved by the scan. Separate palps of the labium are not apparent in the extant larva [9] (supplementary p. 3), and the same is the case in the fossil, as these may be part of the beak structure.

Another clear difference from the fossil is the integration of the mouthparts and the head capsule. The labrum and clypeus are not integrated into the beak in Jurasaidae, while the mentum and stipes form a simple continuum with the head capsule in the latter. While there is only a weak separation of the proximal part of the maxilla from the labium and the head capsule in the fossil on the ventral side, the head capsule, clypeus, and labrum have a clear separation. The labrum is integrated into the beak in the fossil.

In Rosa et al. [9], Jurasaidae and Cerophytidae were resolved as sister groups [9] (figure 6 p. 5). While not as apparent as in Jurasaidae, the larvae of Cerophytidae also have forward-directed protruding mouthparts [20] (figure 10G,H p. 384). They likewise form a beak, although there are clear structural differences. Also, this beak is not as distinct as in *J. itajubense*. It is also broader, in this aspect comparable to the other known larva of Jurasaidae, that of *Tujamita plenalatum* [9] (supplementary figure 12i,j). Also, larvae of Cerophytidae have elongated mandibles [20] (figure 11A,B p. 384), similar to those in Jurasaidae [9] (supplementary figure 12e). The maxillae have a single projecting endite interpreted as mala [20] (p. 381). While the endite appears relatively short in the fossil, this is likely again a matter of preservation—the longer part of the endite reaching into the beak can simply not be resolved, similar to the case of the mandible. In larvae of Cerophytidae, clypeus, labrum, labium, and maxillae are well delineated. Larvae of Cerophytidae have palps on the labium, unlike those of Jurasaidae (although certain setae indicate that these are integrated in the beak) and the fossil. However, they are short, held close to the other parts (as already reported by Costa et al. [20] (p. 382)), and remain indistinct in most directions of view. This may be a precursor condition to that seen in Jurasaidae, where the palps seem to be integrated into the beak.

Overall, we can recognise many similarities between the fossil and the larvae of Cerophytidae and Jurasaidae. Due to these very unusual types of morphologies (with strongly drawn out forward-directed mouthparts forming channels), we see it as likely that the fossil larva (and, in consequence, *Partisaniferus*) is closely related to Cerophytidae and Jurasaidae. In detail, it appears that the morphology of the mouthparts is intermediate between the more plesiomorphic condition in Cerophytidae and the more derived condition in Jurasaidae. We suggest a position of *Partisaniferus* within an unnamed group Cerophytidae + Jurasaidae. This is also consistent with other characters. The very long physogastric trunk region in larvae (and females) of Jurasaidae appears very similar to the condition in two specimens of the beak larva, while the trunk region in larvae of Cerophytidae is also rather soft, but appears less derived. In this aspect, it is interesting that the first beak larva (the single specimen of *P. atrickmuelleri*) has some sclerites on the ventral side that represent dissolved sternites [3], not unlike in larvae of Cerophytidae [20]. However, in the latter, the tergites are still intact, but they are dissolved, i.e., further derived, in *P. atrickmuelleri*.

While we can well understand the channels within the beak through the comparison with Jurasaidae, the channels apparent in the maxillary palps are less easy to understand. These could represent ducts of a large gland. Glands connected to the maxilla are well known in adult and larval beetles (e.g., [30,31,32,33]) but usually end in the proximal region of the maxilla. Small gland ducts have been found also on the palps in some beetles [34], but so far we could not identify a case in the extant fauna with similarly prominent channels. For the moment, the nature and function of these remain unclear.

### 4.3. Lifestyle

It had already been suggested that the beak larva was feeding on fungi and was wood-associated. This seems to be supported by the newly proposed relationships, as this lifestyle is also found in larvae of Cerophytidae [35,36,37]. Such a lifestyle is likely the plesiomorphic condition for Cerophytidae + Jurasaidae, while living in the upper layers of the soil in larvae of Jurasaidae is presumably a derived condition. As other early-branching representatives of Elateroidea are wood-associated (Eucnemidae [38]; Throscidae [39]), these indicate that inhabiting wood is the ancestral condition.

### 4.4. Taxonomical Issues

There are seven species known for Jurasaidae, all extant [9,40,41,42]. In total, 23 extant species are known for Cerophytidae [21,43], but in addition, about the same number of fossil species are known [22,35]. The specimens of the beak larva have been formally described as two species of *Partisaniferus*. The supposed relationship discussed above provides several severe challenges for a taxonomic interpretation of it.

The first aspect comes from the challenge of conspecifity, which not only applies to fossils, but also to extant representatives. We pointed out that it seems that Cerophytidae retained more plesiomorphic characters in the larvae. While this does not immediately transfer to the adults, it is possible that the fossil adults that look similar to modern representatives of Cerophytidae are in fact closer related to Jurasaidae (yet, lacking most of of their apomorphic characters, as common for fossil relatives) and represent the adult of one of the beak larvae. Also, the largest of the beak larva specimens could in fact be a larviform female, which might likewise be conspecific with one of the described species interpreted as a representative of Cerophytidae; still, this latter case is unlikely as even the strongly paedomorphic females of Jurasaidae do not retain their larval beak [9] (supplementary figure 3a,b). For finding further support for any of these possible relationships it would be necessary to find a male in copula with a larviform female, or a larval exuvia attached to a pupa and a pupa with a pharate or freshly emerged adult.

The second aspect is more general and is not related to the problem of fossils but of ranked taxonomy in general. The rank family was given to Jurasaidae. As such decisions are expressions of opinions, it could also have received a subfamily status within Cerophytidae, and the diagnosis for Cerophytidae could have been amended. In the analysis of Rosa et al. [9], only a single species of Cerophytidae was included, making it possible that even the extant species of Cerophytidae are stacked consecutively towards Jurasaidae, possibly either rendering Cerophytidae non-monophyletic (which would demand even more family names) or leading to the one ingroup being reconsidered as Jurasainae, as suggested above.

As a relatively recent example for a comparable case, Jones [44] and Machado et al. [45] both independently recognised that the two supposed sister groups, Ascalaphidae and Myrmeleontidae, in fact have a much more complex phylogenetic relationship. Although resolving very similar phylogenetic trees, they came to drastically differing taxonomic interpretations: Jones [44] increased the number of families of these lacewings to five, while Machado et al. [45] subsummarised everything into a single family Myrmeleontidae, with Ascalaphidae (then called Ascalaphinae) as an ingroup. Both interpretations reflect the phylogeny, although they differ in the taxonomy, demonstrating that what is a family and what is not is arbitrary (see also the discussion in Haug [46]).

It is also not easily possible to erect a name for a monophyletic group that includes what is now considered Jurasaidae, Cerophytidae, and *Partisaniferus*, as the names currently used are all ranked. Also, *Partisaniferus* could be included within Jurasaidae as sister species to the rest; if this idea is not followed, it would be demanded by many authors to erect a distinct monotypic family which would create another (unnecessary) name. There is also no “free” level between the family level and Elateroidea that would be necessary to name the suggested grouping in this frame.

It needs to be emphasised that there can be no correct decision about when something is a family or not. Yet, it is possible to choose less vulnerable naming strategies without endangering names to be changed later. Currently, it seems most useful to not change any of the taxonomic ranks and suggest that the relationships are Cerophytidae + (*Partisaniferus* + Jurasaidae).

### 4.5. Evolution of Ontogeny in Cerophytidae + (Partisaniferus + Jurasaidae)

So far, we know only late stage larvae of Jurasaidae and Cerophytidae. For *Partisaniferus*, however, we also know two specimens of supposed early larval stages. At least for *P. edjarzembowskii*, we can recognise that the early larva is much less physogastric and could represent a functional equivalent to a triungulin larva, a mobile early larva present in several beetles (e.g., [47,48,49,50]). Possibly, at this developmental stage, the mobile larva searched for an entrance into the wood, where it entered and later became physogastric.

As already hypothesised by Haug et al. [8], the beak larva could have been hypermetamorphic. Assuming that the ancestral morphology for Cerophytidae + (*Partisaniferus* + Jurasaidae) was similar to that of *Partisaniferus* means that the early larva was a triungulin-like mobile one. The late larva in Jurasaidae is extremely physogastric, and the body is even more elongated than in the beak larva. The late larva of Cerophytidae is also very soft and bulging, but not strongly elongated. This pattern indicates that the case is most drastic in Jurasaidae. This relative growth is comparable to some representatives of lacewings (e.g., Berothidae [51]), for which hypermetamorphosis has been proposed [52]. This provides already qualitative indications that at least for Jurasaidae the ontogeny is hypermetamorphic.

If we look at quantitative traits, such as body length vs. width ratio (Figure 3H), we can recognise that basically all adults (fossil and extant), the early stage larvae (fossils) and the late larvae of Cerophytidae are comparable in this aspect. Yet, the late larvae of Jurasaidae are more elongated, also providing a quantitative aspect of strongly indirect development (elongating strongly from early larva to late larva, then becoming shorter again in the adult).

The differences between the early and late larvae are less expressed in the beak larvae, as the late larvae appear less derived in body shape. However, we can also recognise indications of hypermetamorphosis for the beak larvae and even for Cerophytidae. In both, the late larvae have quite stout leg elements (Figure 3E–G; [20] (figure 10C,E p. 384); [36] (figure 1B,C p. 21)). These are stouter than those of the early beak larva, where this aspect is accessible [3] (figure 4A p. 13), and stouter than in the adults. Also, late larvae of Jurasaidae have stouter legs [9] (supplementary figure 10g). This developmental pattern from more elongated legs to stouter ones and then to even more elongated ones is also known in species that are considered hypermetamorphic (e.g., in mantis lacewings [53] (figure 4A,B p. 12)).

In this aspect, it is noteworthy that the females in Jurasaidae are strongly paedomorphic, not undergoing the extreme metamorphosis seen in the males [9]. Hence, there are species in which one sex undergoes extreme ontogenetic change (i.e., hypermetamorphosis), while the other sex undergoes relatively little change. This combination leads to even more morphological diversity within a single species. For a “normal” holometabolan, we expect two very distinct ecologies and, coupled with this, morphologies: one for the larvae and one for the adults (as the pupa mostly does not interact much with the environment). For hypermetamorphic species, we expect three of these distinct ecologies and morphologies: early larva, late larva, and adult. For the case of hypermetamorphic species with paedomorphic females, we should have four: early larva, late larva, adult male, and paedomorphic adult female. While we could expect that the adult females are similar to the late larvae, the case in Jurasaidae shows that these females indeed do not have the mouthparts of the larvae, but yet another type of mouthpart [9], reminiscent of those of other larvae of Elateroidea.

### 4.6. Convergence

The elongated larvae in Jurasaidae resemble, in certain aspects, some other larvae of Elateroidea, at least in their worm-like shape. The larvae of many representatives of Eucnemidae are also worm-like, but the later larvae even lack legs entirely [38,54,55]. These worm-like larvae also seem to feed on fungi, but have a very different head shape. Yet, otherwise, these body shapes are rather unique within Elateroidea and therefore represent a case of convergence between Jurasaidae and Eucnemidae.

The head with the strongly protruding mouthparts that characterise the larvae of Cerophytidae + (*Partisaniferus* + Jurasaidae) is very unusual. As pointed out, only larvae of Cerylonidae also have beak-like mouthparts, but no forward-projecting ones [5]. Yet, even outside Holometabola, immatures rarely have beak-like forward-directed mouthparts, as, for example, in extreme cases of hemipterans; however, in these cases, they also face at most antero-ventrally, but not really forward. The immature stages of the extinct palaeodictyopteroideans have such mouthparts [56], most extreme possibly in the larva of *Bizarrea obscura* [57]. Yet, these are in a different size range and therefore likely had quite a different ecology; still, the forward-directed beak is a case of convergence.

### 4.7. Weird Larvae in the Cretaceous

Among fossil larvae are sometimes morphologies that are unknown in the modern fauna, i.e., these seem to have become extinct [46,58,59]. So far, larvae of beetles have not represented strongly expressed cases of such extinct morphologies (indications in [60,61]). The beak larvae, now identified as beetles, may represent such a case. However, the larvae of Jurasaidae seem even more extreme in their morphologies than those of *Partisaniferus edjarzembowskii*. The larva of *P. atrickmuelleri* could still be such a case. Within Elateroidea, dorsal processes in larvae are known, for example, in Lycidae [62] [63] (figure 2E p. 913) [64], Lampyridae [65,66], or Drilini [67] (figure 3k p. 167), although these seem not jointed at all or at least not similar to the processes in *P. atrickmulleri*. Still, the combination of processes with the mouthparts could represent a now-extinct type of morphology among beetle larvae.

## Data Availability

The original contributions presented in this study are included in the article/Supplementary Material. Further inquiries can be directed to the corresponding author.

## Supplementary Materials

The following supporting information can be downloaded at: https://www.mdpi.com/article/10.3390/insects17030316/s1, Supplementary Table S1: Information on the specimens measured from literature data including the ratio of body length to body width.
